# Recirculating hyperthermic intravesical chemotherapy with mitomycin C (HIVEC) versus BCG in high-risk non-muscle-invasive bladder cancer: results of the HIVEC-HR randomized clinical trial

**DOI:** 10.1007/s00345-022-03928-1

**Published:** 2022-01-17

**Authors:** Félix Guerrero-Ramos, Daniel A. González-Padilla, Alejandro González-Díaz, Federico de la Rosa-Kehrmann, Alfredo Rodríguez-Antolín, Brant A. Inman, Felipe Villacampa-Aubá

**Affiliations:** 1 Department of Urology, Hospital Universitario 12 de Octubre, Avenida de Córdoba s/n, 28041 Madrid, Spain; 2 Division of Urology, Duke University Medical Center, Durham, NC USA; 3 Department of Urology, Clínica Universidad de Navarra, Madrid, Spain

**Keywords:** Bladder cancer, Bacillus Calmette–Guérin, Hyperthermia, Recurrence, Progression

## Abstract

**Purpose:**

The purpose of the study was to compare the outcomes of high-risk non-muscle-invasive bladder cancer (HR-NMIBC) patients treated with BCG vs recirculating hyperthermic intravesical chemotherapy (HIVEC) with mitomycin C (MMC).

**Methods:**

A pilot phase II randomized clinical trial was conducted including HR-NMIBC patients, excluding carcinoma in situ. Patients were randomized 1:1 to receive intravesical BCG for 1 year (once weekly for 6 weeks plus subsequent maintenance) or HIVEC with 40 mg MMC, administered using the Combat BRS system (once weekly instillations were given for 6 weeks, followed by once monthly instillation for 6 months). Total recirculating dwell time for HIVEC was 60 min at a target temperature of 43° ± 0.5 °C. Primary endpoint was recurrence-free survival. Secondary endpoints were time to recurrence, progression-free survival, cancer-specific survival, and overall survival at 24 months. Adverse events were routinely assessed.

**Results:**

Fifty patients were enrolled. Mean age was 73.5 years. Median follow-up was 33.7 months. Recurrence-free survival at 24 months was 86.5% for HIVEC and 71.8% for BCG (*p* = 0.184) in the intention-to-treat analysis and 95.0% for HIVEC and 75.1% for BCG (*p* = 0.064) in the per protocol analysis. Time to recurrence was 21.5 and 16.1 months for HIVEC and BCG, respectively. Progression-free survival for HIVEC vs BCG was 95.7% vs 71.8% (*p* = 0.043) in the intention-to-treat analysis and 100% vs 75.1% (*p* = 0.018) in the per protocol analysis, respectively. Cancer-specific survival at 24 months was 100% for both groups and overall survival was 91.5% for HIVEC vs 81.8% for BCG.

**Conclusion:**

HIVEC provides comparable safety and efficacy to BCG and is a reasonable alternative during BCG shortages.

**Trial registration:**

EudraCT 2016-001186-85. Date of registration: 17 March 2016.

**Supplementary Information:**

The online version contains supplementary material available at 10.1007/s00345-022-03928-1.

## Introduction

The high rates of recurrence and progression—reaching 60–80% and 20–40% at 5 years, respectively—are hallmarks of high-risk non-muscle-invasive bladder cancer (NMIBC) [1, 2]. Several clinical trials have found intravesical bacillus Calmette–Guérin (BCG) treatment with maintenance to be the best bladder-sparing treatment for these high-risk NMIBC patients [3–5]. However, the administration of BCG is not without toxicity, with 63% of patients reporting local side effects and 31% reporting systemic side effects [6]. Additionally, recent severe global shortages in BCG supply have compromised patient outcomes and left clinicians around the globe scrambling to identify effective and reliable alternative therapies [7–9].

Hyperthermic intravesical chemotherapy (HIVEC) has shown promise as an alternative to BCG [10]. Key mechanistic benefits of HIVEC over standard room temperature intravesical chemotherapy are improved drug delivery and penetration into the bladder, improved chemotherapy efficacy in heated cancer cells and the triggering of local anticancer immune reactions [11]. Different technologies can be used to achieve bladder heating [12]. Here, we present the results of HIVEC-HR, the first randomized clinical trial of recirculating convective HIVEC using mitomycin C (MMC) compared to BCG in patients with high-risk papillary NMIBC.

## Materials and methods

### Study design and participants

HIVEC-HR is a pilot phase 2 randomized clinical trial conducted between November 2016 and March 2021. Eligible patients had a diagnosis of high-risk papillary NMIBC as defined by the 2016 version of the EAU guidelines. Patients were excluded if they had carcinoma in situ (CIS), a history of hypersensitivity or allergy to MMC, a history of BCG intolerance, or any condition that contraindicated the administration of BCG. Prior intravesical therapy was permitted.

The clinical trial was designed and implemented by the Department of Urology at University Hospital 12 Octubre, Madrid, meeting the criteria for an investigator-initiated study. The study protocol and informed consent were approved by the Spanish Agency for Medicines and Health Products (AEMPS) and local Ethics Committees.

### Study rationale

This pilot clinical trial was designed during a global BCG shortage, when 3 years’ therapy was not feasible due to supply restrictions. Alternative therapies were explored in the context of a clinical trial setting. CIS was excluded due to lack of evidence on the use of hyperthermic instillations in this context.

### Procedures

Prior to enrollment, all visible tumors were resected (TURBT) under augmented endoscopic vision. Re-TURBT was done 2–6 weeks after first TURBT in six BCG patients and six HIVEC patients. The criteria for re-TURBT were incomplete resection, absence of muscularis mucosae in the specimen (Tx), or inability to evaluate tumor infiltration. Consenting patients were randomized with a 1:1 allocation ratio to receive HIVEC or BCG. HIVEC was administered using the Combat BRS system (Combat Medical Ltd, Wheathampstead, UK) combined with 40 mg MMC diluted in 40 mL distilled water. Total recirculating dwell time for HIVEC was 60 min at a target temperature of 43° ± 0.5 °C. HIVEC treatment consisted of induction (once weekly instillations for 6 weeks) followed by maintenance (once monthly instillations for 6 months). For BCG therapy, 50 mg OncoTICE^®^ was administered once weekly for 6 weeks (induction) and subsequently once weekly for 3 weeks at 3, 6, and 12 months (maintenance). Adjuvant therapy in both groups started at 40 days postoperatively.

Bladder surveillance for tumor recurrence consisted of urine cytology and cystoscopy at 3, 6, 9, 12, 15, 18, and 24 months. Abdominal and pelvic computerized tomography urography was performed at the screening visit and then yearly or if clinically indicated. TURBT was performed if urine cytology was positive, cystoscopy was abnormal, or imaging suggested a possible cancer recurrence.

### Outcomes

The primary endpoint for the trial was recurrence-free survival (RFS) following TURBT. Secondary endpoints included time to recurrence (TTR), progression-free survival (PFS), cancer-specific survival (CSS), and overall survival (OS) at 24 months. Adverse events (AEs) were routinely assessed according to Common Terminology Criteria for Adverse Events (CTCAE) version 4.0.

### Statistical analysis

Since the efficacy of HIVEC in high-risk NMIBC patients was not known when this study was planned, this trial was designed as a pilot study with a convenient sample size of 50 (25 per arm) to inform future larger randomized trials. Using the CUETO nomogram [2], the 24-month RFS for a high-risk NMIBC study population treated with BCG was expected to be approximately 50%. With a one-sided, two-sample test for proportions with alpha at 0.1, we estimated that the power to detect a 50% improvement in 24-month RFS (0.75) with HIVEC was 71%. For efficacy evaluation, two study populations were established: the intention-to-treat (ITT) population, defined as any randomized patient, and the per protocol (PP) population, defined as any randomized patient who completed induction therapy and met the eligibility criteria (one patient was diagnosed with CIS at re-review and was excluded from the PP population despite completed induction). Reasons for not receiving the intended treatment are listed in Supplementary Table 1.

For efficacy evaluation, the Kaplan–Meier survivor function was used and compared using the Wilcoxon test. Univariate Cox regression models were obtained for RFS and PFS. The hazard ratio (HR) and its 95% confidence interval (CI) were calculated. There were insufficient events to allow for multivariate modeling. All patients who received at least one intravesical instillation were included in the safety analysis. Chi-square and Fisher tests were used to compare AEs between the treatment arms.

## Results

In total, 50 patients were randomized, of whom 48 received at least one treatment session. Reasons for not receiving treatment included a new urethral stricture that prevented bladder catheterization and hospitalization due to pneumonia prior to the first instillation (Supplementary Fig. 1). Baseline patient characteristics are shown in Table 1**.** Mean patient age was 73.5 years and 88% were male. Median follow-up was 33.7 months (interquartile interval: 18.6–37.1). There were 11 recurrences (BCG = 7, HIVEC = 4), 7 disease progressions (BCG = 6, HIVEC = 1), and 11 deaths (BCG = 7, HIVEC = 4) during the trial, 2 of which were due to bladder cancer.

**Table 1:** Baseline characteristics of participants in both treatment arms

Baseline characteristics	BCG		HIVEC	
	*n*	%	*n*	%
Gender				
Men	22	88	21	84
Women	3	12	4	16
Age				
Mean (± SD)	73.0 ± 8.65		74.1 ± 10.4	
Primary	18	72	22	88
Primary vs recurrent				
Recurrent < 1/year	3	12	3	12
Recurrent > 1/year	4	16	0	0
Stage				
Ta	11	44	14	56
T1	14	56	11	44
Grade				
G2	0	0	1	4
G3	25	100	24	96
Number of tumors				
1	19	76	19	76
2–7	5	20	6	24
≥ 8	1	4	0	0
Tumor size				
< 3 cm	17	68	15	60
≥ 3 cm	8	32	10	40
Postoperative MMC				
No	18	72	20	80
Yes	7	28	5	20
Prior intravesical therapy				
No	20	80	23	92
Yes	5*	20	0	0
Unknown	0	0	2	8
Repeat second-look TURBT				
No	19	76	19	76
Yes	6	24	6	24

Five patients in the BCG arm had received previous intravesical therapy, four with MMC and one with BCG

*BCG* Bacillus Calmette–Guérin, *HIVEC* hyperthermic intravesical chemotherapy, *MMC* mitomycin C, *TURBT* transurethral resection of bladder tumor

**Fig. 1:**
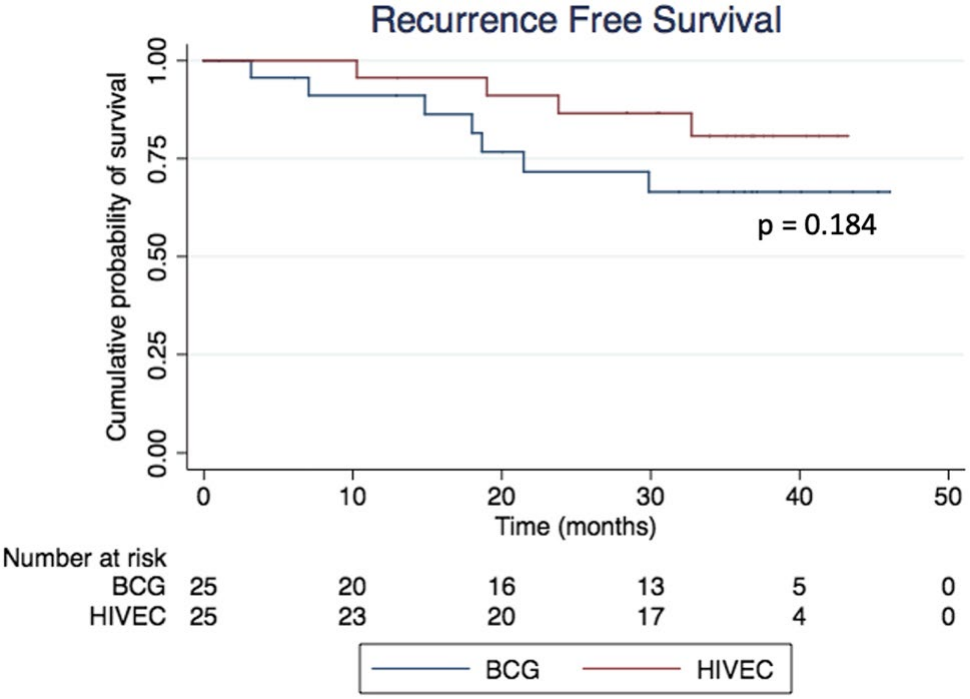
Recurrence-free survival (RFS) curves in both treatment groups—ITT analysis

**Fig. 2:**
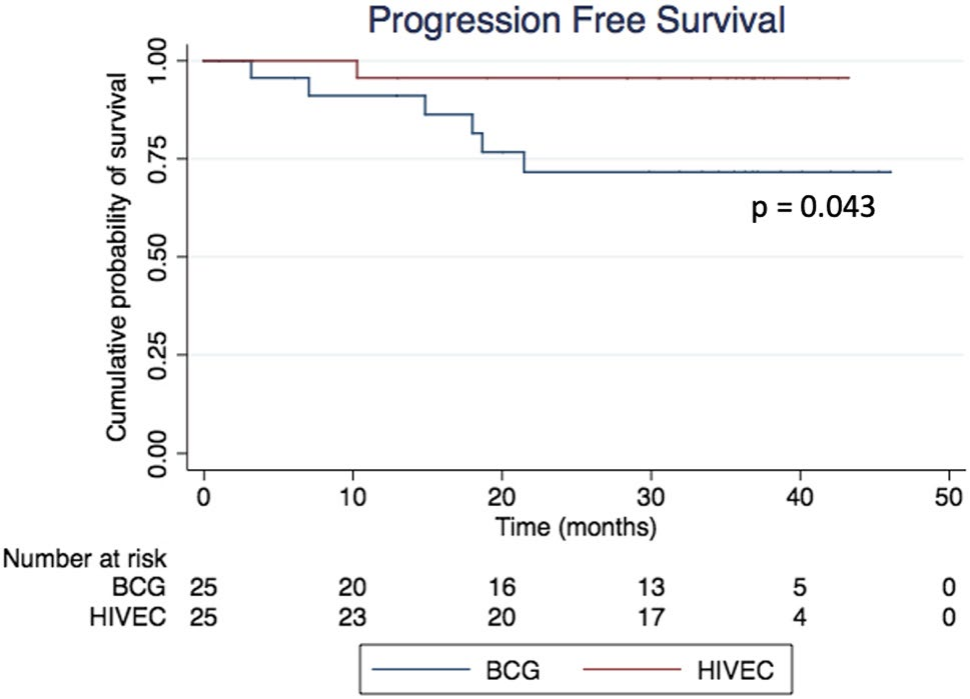
Progression-free survival (PFS) curves in both treatment groups—ITT analysis

In the ITT analysis, the 24-month RFS was 86.5% for HIVEC and 71.8% for BCG (HR 0.41, 95% CI 0.10–1.66; *p* = 0.215); the corresponding values in the PP analysis were 95.0% and 75.1%, respectively (HR 0.48; 95% CI 0.11–2.03; *p* = 0.322). Mean TTR with HIVEC was 21.5 months, while with BCG it was 16.1 months (*p* = 0.315). The ITT 24-month PFS was 95.7% for HIVEC and 71.8% for BCG (HR 0.14, 95% CI 0.02–1.29; *p* = 0.071), while in the PP analysis it was 100% for HIVEC and 75.1% for BCG (HR 0.16, 95% CI 0.02–1.4; *p* = 0.102) (Figs. 1 and 2). At 24 months, CSS was 100% in both treatment groups, and the 24-month OS was similar (91.5% for HIVEC vs 81.8% for BCG, *p* = 0.498). The cystectomy rate was 4% in the HIVEC arm and 20% in the BCG arm. Cox regression analysis showed the ITT hazard ratios for HIVEC vs BCG for the RFS, PFS, and OS endpoints to be 0.47 (95% CI 0.13–1.61), 0.14 (95% CI 0.02–1.19), and 0.75 (95% CI 0.23–2.48), respectively.

A total of 31 patients (64.6%) reported at least one AE and 23 (47.9%) reported at least one study therapy-related AE (Table 2). There were two patients with grade 4 or 5 study therapy-related AEs, both in the BCG arm. One patient developed urosepsis requiring hospital admission and one died as a consequence of Guillain–Barré syndrome, which was deemed to be treatment related (Supplementary Table 2). The overall rate and grade of study therapy-related AEs were similar between the arms, with slightly more grade ≥ 3 complications in the BCG arm. Two HIVEC patients who developed MMC allergy during induction received monthly maintenance BCG for 6 months. Treatment was discontinued due to study therapy-related AEs in four HIVEC patients and seven BCG patients. Regarding grade 1 and 2 study therapy-related AEs, bladder spasms were more frequent in the HIVEC group. Supplementary Table 3 shows the grading, according to CTCAE (Common Terminology Criteria for Adverse Events) version 4, of the AEs in relation to the treatment.

**Table 2:** Description of study therapy-related adverse events

Study therapy-related adverse events	Grade 1–2		Grade 3		Grade 4–5	
	HIVEC	BCG	HIVEC	BCG	HIVEC	BCG
Hematuria	1 (4.2%)	1 (4.2%)		1 (4.2%)		
Irritative		1 (4.2%)	1 (4.2%)			
Spasms	7 (29.2%)					
Fever				3 (12.5%)		
UTI		2 (8.3%)				
Allergy			3 (12.5%)			
Dysuria		1 (4.2%)				
Other						2 (8.3%)
Total	8 (33.3%)	5 (20.8%)	4 (16.7%)	4 (16.7%)	0	2 (8.3%)

The safety analysis was performed in all randomized patients who received at least one instillation (*n* = 48)

*UTI* urinary tract infection

## Discussion

We have presented results from HIVEC-HR, the first randomized clinical trial comparing recirculating conductive HIVEC and BCG in patients with high-risk papillary NMIBC. The results suggest that HIVEC with MMC induction plus maintenance appears at least comparable to BCG in terms of safety and efficacy. HIVEC did not show any evidence of being worse than BCG with respect to any of the clinical efficacy endpoints. From a safety perspective, we found that both treatments had similar AE profiles to those reported elsewhere [6]. The results of our work are particularly relevant in the current health care environment, in which BCG has limited availability and alternative therapies are needed. Indeed, centers in Europe and North American have implemented conductive HIVEC when BCG is not available [13–15].

While this is the first randomized trial to use the Combat BRS system to administer HIVEC^®^ treatment with MMC in high-risk NMIBC, other bladder-heating devices have been tested in this population. Arends et al*.* compared heated MMC using the Synergo SB-TS 101 device with BCG in 190 intermediate- and high-risk NMIBC patients [16] and found that heated MMC was superior to BCG in patients with papillary tumors (24 month RFS: 78% vs 65%). A second randomized trial similarly compared Synergo-heated MMC with BCG in 104 patients with intermediate- and high-risk NMIBC [17]. Patients with CIS had a worse RFS with heated MMC (HR = 2.06), while those with papillary tumors had a better RFS (HR = 0.50). When our trial was designed, CIS was considered an exclusion criterion based on the aforementioned data. However, currently most of the new drugs for NMIBC are being tested in the BCG-unresponsive population, with promising outcomes for immune checkpoint inhibitors in CIS and for intravesical therapies such as nadofaragene firadenovec in both CIS and papillary neoplasms [18, 19]. Recent data from a retrospective analysis using HIVEC for a mixed BCG-unresponsive population are also encouraging with the added benefit of a better safety profile as compared with systemic therapies [20].

While HIVEC-HR has many strengths, it also has weaknesses. First, the results we present were derived from a phase II pilot trial in which RFS was the main objective (a pilot study is a small-scale exploratory study that tries to answer questions such as the pragmatics of recruitment and whether a larger trial is feasible). Despite this being a phase II trial, RFS was set as the primary outcome due to the urgent need for efficacy data for alternative treatments to BCG in the context of the severe BCG shortage, and this is how the trial was approved by the Regulatory Agencies and Ethics Committees. Despite the value of a pilot study, it has its own limitations, particularly a small sample size. Second, five patients in the BCG group had failed an intravesical treatment before this trial whereas only two patients in the HIVEC group had prior intravesical treatment. Because of the pilot design, the number of patients and the number of events are low, so these differences may have had a significant confounding impact on the results. Third, while the results are interestingly good in favor of HIVEC, the duration of response might be the subject of further evaluation with a longer follow-up.

HIVEC-HR did not mandate repeat TURBT and immediate postoperative chemotherapy and, while there was no significant difference between the arms with respect to these two interventions, future trials should specify their requirement.

## Conclusions

HIVEC-HR compared HIVEC and BCG in patients with high-risk papillary NMIBC. This pilot trial suggests that HIVEC with MMC provides comparable safety and efficacy to BCG and represents a reasonable alternative that should be considered during BCG shortages. A larger trial will be required to determine whether HIVEC is superior to BCG in this population.

## Supplementary Information

Below is the link to the electronic supplementary material.

Supplementary file1 (DOCX 14 KB)

Supplementary file2 (DOCX 34 KB)

Supplementary file3 (DOCX 15 KB)

Supplementary file4 (DOCX 60 KB)
