# Supplementary material for: Recirculating hyperthermic intravesical chemotherapy with mitomycin C (HIVEC) versus BCG in high-risk non-muscle-invasive bladder cancer: results of the HIVEC-HR randomized clinical trial
Source: World J Urol. 2022 Jan 17;40(4):999–1004. doi: 10.1007/s00345-022-03928-1 (PMC8994727; doi:10.1007/s00345-022-03928-1)
Supplement: Supplementary file 1 — Supplementary file1 (DOCX 14 KB) [file 345_2022_3928_MOESM1_ESM.docx]

| **Treatment arm** | **Number of instillations received** | **Cause of discontinuation** | **Status / recurrence / progression** |
| --- | --- | --- | --- |
| BCG | 0 | Uretheral stenosis | Exitus due to gastric neoplastic disease |
| HIVEC | 0 | Hospitalization due to pneumonia | Exitus due to acute myocardial infarction |
| HIVEC | 4 | MMC allergy | Progression to T2G3; neo + cystectomy T0N0M0 |
| BCG | 6+3 | Concomitant CIS | Progression to T2G3; cystectomy T4N2M0. Exitus due to bone metastases |
| HIVEC | 5 | MMC allergy (continued with BCG × 6) | Recurrence TURBT – TaG3 treated with BCG |
| BCG | 4 | Fever | Exitus due to lung cancer |
| BCG | 5 | Guillain-Barré syndrome | Exitus due to Guillain-Barré syndrome |

Supplementary Table 1. Reasons for exclusion from the per protocol (PP) population, defined as any randomized patient who completed induction therapy and met the eligibility criteria

HIVEC, Hyperthermic intravesical chemotherapy; BCG, bacillus Calmette-Guérin
