# Supplementary material for: Recirculating hyperthermic intravesical chemotherapy with mitomycin C (HIVEC) versus BCG in high-risk non-muscle-invasive bladder cancer: results of the HIVEC-HR randomized clinical trial
Source: World J Urol. 2022 Jan 17;40(4):999–1004. doi: 10.1007/s00345-022-03928-1 (PMC8994727; doi:10.1007/s00345-022-03928-1)
Supplement: Supplementary file 2 — Supplementary file2 (DOCX 34 KB) [file 345_2022_3928_MOESM2_ESM.docx]

**Supplementary Table 2.** Details of all adverse events in both treatment groups, and their consequences*

| AE no. | Patient age (yr) | Treatment | Instillations  (i + m) | AE | CTCAE grade | Consequence / status | Relation to therapy |
| --- | --- | --- | --- | --- | --- | --- | --- |
| 1 | 84 | HIVEC | 6 + 2 | Hematuria | 1 | None / resolved | Related |
| 2 | 68 | BCG | 6 + 9 | Irritative symptoms | 2 | Delayed instillation / resolved | Related |
| 3 | 68 | BCG | 6 + 9 | Covid-19 | 5 | Exitus (41 m after enrolment) | Not related |
| 4 | 66 | HIVEC | 6 + 5 | Bladder spasms | 1 | None / resolved | Related |
| 5 | 73 | HIVEC | 6 + 6 | Bladder spasms | 1 | None / resolved | Related |
| 6 | 62 | BCG | 6 + 9 | Rectal neoplasm | 3 | Concomitant medication / resolved | Not related |
| 7 | 76 | HIVEC | 6 + 6 | Rectal neoplasm, metastases | 5 | Concomitant medication / exitus | Not related |
| 8 | 71 | BCG | 6 + 2 | Fever | 3 | Therapy discontinuation / resolved | Related |
| 9 | 64 | HIVEC | 6 + 5 | Irritative symptoms | 3 | Therapy discontinuation / resolved | Related |
| 10 | 76 | BCG | 6 + 0 | Hematuria | 3 | Therapy discontinuation / resolved | Related |
| 11 | 76 | BCG | 6 + 0 | Progression T2AG + CIS | 3 | Cystectomy / resolved | Related |
| 12 | 74 | HIVEC | 6 + 6 | Bladder spasms | 1 | None / resolved | Related |
| 13 | 86 | BCG | 6 + 4 | Hematuria | 2 | None / resolved | Related |
| 14 | 86 | BCG | 6+4 | Renal dysfunction | 3 | Therapy discontinuation / resolved | Not related |
| 15 | 86 | BCG | 6 + 4 | Colon Ca | 5 | Exitus | Not related |
| 16 | 62 | HIVEC | 6 + 6 | Bladder spasms | 1 | None / resolved | Related |
| 17 | 68 | BCG | 6 + 9 | Dysuria | 2 | Concomitant medication / resolved | Related |
| 18 | 68 | BCG | 6 + 9 | pT3bN2M0 | 4 | Cystectomy + Bricker / Oncology unit, metastases | Related |
| 19 | 82 | HIVEC | 6 + 6 | Bladder spasms | 1 | None / resolved | Related |
| 20 | 80 | BCG | 6 + 9 | UTI | 2 | Concomitant medication / resolved | Related |
| 21 | 80 | BCG | 6 + 9 | pT2G3cT4 | 5 | Neo + cystectomy / exitus | Related |
| 22 | 73 | HIVEC | 6 + 6 | Bladder spasms | 2 | Concomitant medication / resolved | Related |
| 23 | 57 | HIVEC | 4 + 0 | MMC allergy | 3 | Therapy discontinuation / resolved | Related |
| 24 | 57 | HIVEC | 4 + 0 | pT2G3 | 3 | Neo + cystectomy / resolved | Related |
| 25 | 80 | HIVEC | 6 + 6 | Bladder spasms | 1 | None / resolved | Related |
| 26 | 80 | HIVEC | 6 + 6 | Rectal neoplasm IV | 5 | Exitus | Not related |
| 27 | 72 | BCG | 6 + 3 | T4N2M0 | 5 | Exitus | Related (had CIS at randomization) |
| 28 | 89 | BCG | 6 + 2 | Fever | 3 | Therapy discontinuation / resolved | Related |
| 29 | 71 | BCG | 6 + 9 | pT2bN0M0R1 | 4 | Neo + cystectomy / resolved | Related |
| 30 | 90 | BCG | 6 + 9 | pT4 and M1 | 4 | Palliative unit | Related |
| 31 | 69 | BCG | 6 + 9 | UTI | 2 | Concomitant medication / resolved | Related |
| 32 | 69 | BCG | 6 + 0 | Pancreatic adenocarcinoma | 5 | Therapy discontinuation / exitus | Not related |
| 33 | 77 | HIVEC | 6 + 6 | Heart failure | 5 | Exitus | Not related |
| 34 | 73 | HIVEC | 6 (HIVEC) + 6 (BCG) | MMC allergy | 3 | Therapy discontinuation / resolved | Related |
| 35 | 85 | HIVEC | 6 + 0 | Ictus | 5 | Exitus | Not related |
| 36 | 82 | BCG | 6 + 7 | UTI | 4 | Therapy discontinuation / resolved | Related |
| 37 | 72 | HIVEC | 5 (HIVEC) + 6 (BCG) | MMC allergy | 3 | Therapy discontinuation / resolved | Related |
| 38 | 87 | BCG | 4 + 0 | Fever | 3 | Therapy discontinuation / resolved | Related |
| 39 | 87 | BCG | 4 + 0 | Lung cancer | 5 | Concomitant medication / exitus | Not related |
| 40 | 83 | BCG | 5 + 0 | Guillain-Barré syndrome | 5 | Therapy discontinuation / exitus | Related |

*Bladder tumor recurrence with no tumor stage progression was not considered an adverse event but was included in the study endpoint assessment

Instillations (i + m), received instillations (induction + maintenance); AE, adverse event; CTCAE, Common Terminology Criteria for Adverse Events; HIVEC, hyperthermic intravesical chemotherapy; BCG, bacillus Calmette-Guérin; UTI, urinary tract infection
