# Supplementary material for: Recirculating hyperthermic intravesical chemotherapy with mitomycin C (HIVEC) versus BCG in high-risk non-muscle-invasive bladder cancer: results of the HIVEC-HR randomized clinical trial
Source: World J Urol. 2022 Jan 17;40(4):999–1004. doi: 10.1007/s00345-022-03928-1 (PMC8994727; doi:10.1007/s00345-022-03928-1)
Supplement: Supplementary file 3 — Supplementary file3 (DOCX 15 KB) [file 345_2022_3928_MOESM3_ESM.docx]

| CTCAE v4.0 | Grade 1 | Grade 2 | Grade 3 | Grade 4 | Grade 5 |
| --- | --- | --- | --- | --- | --- |
| Bladder spasm | Intervention not indicated | Antispasmodic indicated | Hospitalization indicated |  |  |
| Cystitis, non-infective | Microscopic hematuria; minimal increase in frequency, urgency, dysuria, or nocturia; new onset of incontinence | Moderate hematuria; moderate increase in frequency, urgency, dysuria, nocturia, or incontinence; urinary catheter placement or bladder irrigation indicated; limiting instrumental ADL* | Gross hematuria; transfusion, IV medications or hospitalization indicated; elective endoscopic, radiologic, or operative intervention indicated | Life-threatening consequences; urgent radiologic or operative intervention indicated | Death |
| Hematuria | Asymptomatic; clinical or diagnostic observations only; intervention not indicated | Symptomatic; urinary catheter or bladder irrigation indicated; limiting instrumental ADL | Gross hematuria; transfusion, IV medications, or hospitalization indicated; elective endoscopic, radiologic, or operative intervention indicated; limiting self-care ADL** | Life-threatening consequences; urgent radiologic or operative intervention indicated | Death |
| Renal and urinary disorders – other, specify | Asymptomatic or mild symptoms; clinical or diagnostic observations only; intervention not indicated | Moderate, local, or non-invasive intervention indicated; limiting instrumental ADL | Severe or medically significant but not immediately life- threatening; hospitalization or prolongation of existing hospitalization indicated; disabling; limiting self-care ADL | Life-threatening consequences; urgent intervention indicated | Death |
| Fever (General disorders section) | 38.0°–39.0°C | 39.1°–40°C | >40°C for ≤24 h | >40°C for >24 h | Death |

Supplementary Table 3. Grading of AEs caused by instillations, according to CTCAE (Common Terminology Criteria for Adverse Events) version 4

ADL, activities of daily living

*Instrumental ADL refers to preparing meals, shopping for groceries or clothes, using the telephone, managing money, etc.

**Self-care ADL refers to bathing, dressing and undressing, feeding self, using the toilet, taking medications, and not being bedridden.
