# Supplementary material for: Recirculating hyperthermic intravesical chemotherapy with mitomycin C (HIVEC) versus BCG in high-risk non-muscle-invasive bladder cancer: results of the HIVEC-HR randomized clinical trial
Source: World J Urol. 2022 Jan 17;40(4):999–1004. doi: 10.1007/s00345-022-03928-1 (PMC8994727; doi:10.1007/s00345-022-03928-1)
Supplement: Supplementary file 4 — Supplementary file4 (DOCX 60 KB) [file 345_2022_3928_MOESM4_ESM.docx]

Assessed for efficacy in ITT analysis – all randomized patients (n=25)

Assessed for safety – all patients who received at least 1 dose (n=24)

Assessed in PP analysis – patients who met the eligibility criteria and completed induction (n=22)

Lost to follow-up (n=0)

Discontinued intervention (n=4) (3 MMC allergy, 1 irritative symptoms)

Allocated to HIVEC (n=25)

♦ Received allocated intervention (n=24)

♦ Did not receive allocated intervention (pneumonia) (n=1)

Lost to follow-up (n=0)

Discontinued intervention (n=8) (3 fever, 1 renal dysfunction, 1 UTI, 1 hematuria, 1 pancreatic adenocarcinoma, 1 Guillain-Barré syndrome)

Allocated to BCG (n=25)

♦ Received allocated intervention (n=24)

♦ Did not receive allocated intervention (urethral stricture) (n=1)

Assessed for efficacy in ITT analysis – all randomized patients (n=25)

Assessed for safety – all patients who received at least 1 dose (n=24)

Assessed in PP analysis – patients who met the eligibility criteria and completed induction (n=21)

## Allocation

## Assessment

## Intervention

Randomized (n=50)
